# Limited Predictive Value of Inflammatory and Renal Markers in the Progression of Isolated Gestational Proteinuria to Preeclampsia: A Retrospective Cohort Study

**DOI:** 10.3390/jcm15103966

**Published:** 2026-05-21

**Authors:** Dinçer Sümer, Ahmet Arif Filiz, Pelin Yıldırım, Ahsen Bayraktar, İslam Aslanlı, Ayşenur Göksu, Kubilay Çanga, Zehra Vural Yılmaz

**Affiliations:** 1 Department of Perinatology, University of Health Sciences, Etlik City Hospital, Varlık Mahallesi Halil Sezai Erkut Cd. No:5, Yenimahalle, Ankara 06170, Türkiye; ahmetarif_filiz@hotmail.com (A.A.F.); kubilaycanga@hotmail.com (K.Ç.); zehravural@gmail.com (Z.V.Y.); 2 Department of Obstetrics and Gynecology, University of Health Sciences, Etlik City Hospital, Varlık Mahallesi Halil Sezai Erkut Cd. No:5, Yenimahalle, Ankara 06170, Türkiye; yildirimmpelin@hotmail.com (P.Y.); ahsen.bayraktar988@gmail.com (A.B.); islamaslanli80@gmail.com (İ.A.); aysenurgoksu10@gmail.com (A.G.)

**Keywords:** isolated gestational proteinuria, preeclampsia, pregnancy complications, obstetrics outcome

## Abstract

**Objective:** Isolated gestational proteinuria (IGP) has traditionally been considered a benign condition; however, emerging evidence suggests that it may represent an early stage in the spectrum of preeclampsia. This study aimed to evaluate clinical and laboratory predictors of progression from IGP to preeclampsia. **Methods:** This retrospective cohort study included pregnant women diagnosed with proteinuria ≥ 300 mg/day after 20 weeks of gestation between January 2023 and December 2024. After applying predefined exclusion criteria, 319 women with isolated gestational proteinuria (IGP) were included and stratified according to progression to preeclampsia (*n* = 42, 17.8%). Baseline clinical and laboratory parameters were compared between groups. Multivariable logistic regression analysis was performed to identify independent predictors of progression, and receiver operating characteristic (ROC) curve analysis was used to evaluate the discriminative performance of significant variables. **Results:** Preeclampsia developed in 17.8% of women with IGP. In multivariable analysis, higher maternal body mass index (aOR 1.085, *p* = 0.028) and earlier gestational age at diagnosis (aOR 0.883, *p* = 0.011) were identified as independent predictors of progression. Although neutrophil count and systemic inflammatory indices were elevated in univariate analyses, they did not retain independent predictive value after adjustment. **Conclusions:** In pregnancies complicated by isolated gestational proteinuria, clinical parameters appear to be more informative than inflammatory and renal markers for predicting progression to preeclampsia. Laboratory-derived indices offer limited additional value and should be interpreted cautiously in risk assessment.

## 1. Introduction

Hypertensive disorders of pregnancy continue to be a major cause of maternal and perinatal morbidity and mortality worldwide. Preeclampsia, defined by new-onset hypertension with proteinuria or other signs of maternal organ dysfunction after 20 weeks of gestation, is one of the most clinically significant forms of these disorders [1,2]. Despite extensive research, early identification of women at risk for preeclampsia remains challenging.

Isolated gestational proteinuria (IGP) is defined as significant proteinuria occurring after 20 weeks of gestation without hypertension or other diagnostic criteria for preeclampsia. Although traditionally considered a benign or transient finding, accumulating evidence suggests that isolated proteinuria may be an early sign of placental dysfunction and may precede the clinical onset of preeclampsia in a substantial proportion of cases. Previous studies have reported that approximately 20–25% of women with isolated gestational proteinuria later develop preeclampsia, underscoring the need for close monitoring in this population [3,4,5]. Identifying patients with isolated proteinuria who are at increased risk of progressing to preeclampsia is therefore clinically important. Early risk stratification may enable closer surveillance, timely intervention, and improved maternal and neonatal outcomes. In recent years, systemic inflammatory indices derived from routine complete blood count parameters—such as the neutrophil-to-lymphocyte ratio (NLR), platelet-to-lymphocyte ratio (PLR), monocyte-to-lymphocyte ratio (MLR), systemic immune-inflammation index (SII), and systemic inflammation response index (SIRI)—have emerged as potential markers reflecting the inflammatory component of placental dysfunction and hypertensive disorders of pregnancy [6,7,8]. However, data on the predictive value of demographic characteristics and systemic inflammatory markers for progression from isolated proteinuria to preeclampsia remain limited. Given the growing interest in laboratory-derived inflammatory indices in obstetric prediction models, evaluating whether these markers provide additional clinical value beyond conventional clinical parameters is important.

Therefore, the aim of this study was to investigate whether maternal demographic characteristics and systemic inflammatory indices derived Checked and confirmedfrom routine laboratory parameters can predict the progression of isolated gestational proteinuria to preeclampsia.

## 2. Materials and Methods

### 2.1. Study Design

This retrospective study was conducted at the Perinatology Department of Ankara Etlik City Hospital, a large tertiary referral center with a high obstetric volume, and included pregnant women with proteinuria (>300 mg/24 h) after 20 weeks of gestation between January 2023 and December 2024 who subsequently delivered at our institution. During the study period, a total of 25,194 deliveries were recorded at our center. The study was conducted in accordance with the Declaration of Helsinki and approved by the Ethics Committee of Ankara Etlik City Hospital (approval number: AEŞH-BADEK-2025-0098). Due to the retrospective design, the Ethics Committee waived the requirement for informed consent. Patient data were obtained from electronic medical records and the hospital information management system and were analyzed anonymously.

This study was reported in accordance with the STROBE guidelines for observational cohort studies.

### 2.2. Patient Selection

Twenty-four–hour urine protein assessment was performed based on clinical indications rather than universal screening, including cases with positive dipstick proteinuria on routine antenatal screening, suspected hypertensive disorders of pregnancy, or clinical features suggestive of preeclampsia. In these cases, 24 h urine collection was used to quantify protein excretion according to institutional protocols. Twenty-four–hour urine samples were collected in accordance with institutional protocols. Patients were instructed to discard the first morning urine sample and collect all subsequent urine over the next 24 h, including the first sample of the following morning. Total urine volume was recorded, and protein excretion was measured using standard biochemical laboratory methods. Collections with suspected incomplete sampling were excluded from analysis. To minimize potential confounding, urinalysis and urine culture results obtained concurrently with 24 h urine collection were reviewed, and patients with culture-proven urinary tract infection were excluded from analysis. Therefore, the analyzed cohort should be interpreted as a clinically selected higher-risk population rather than a universally screened obstetric cohort.

Patients were excluded if they met any of the following criteria: multiple pregnancy; chronic systemic disease; gestational age outside the specified range; fetal chromosomal or structural anomalies; pregnancies complicated by gestational diabetes mellitus, intrahepatic cholestasis, or other significant obstetric comorbidities; systemic steroid use or known drug allergy; antenatal care performed outside our institution; or a diagnosis of preeclampsia or severe preeclampsia at initial clinical evaluation requiring immediate delivery before completion of 24 h urine protein quantification.

**Figure 1:**
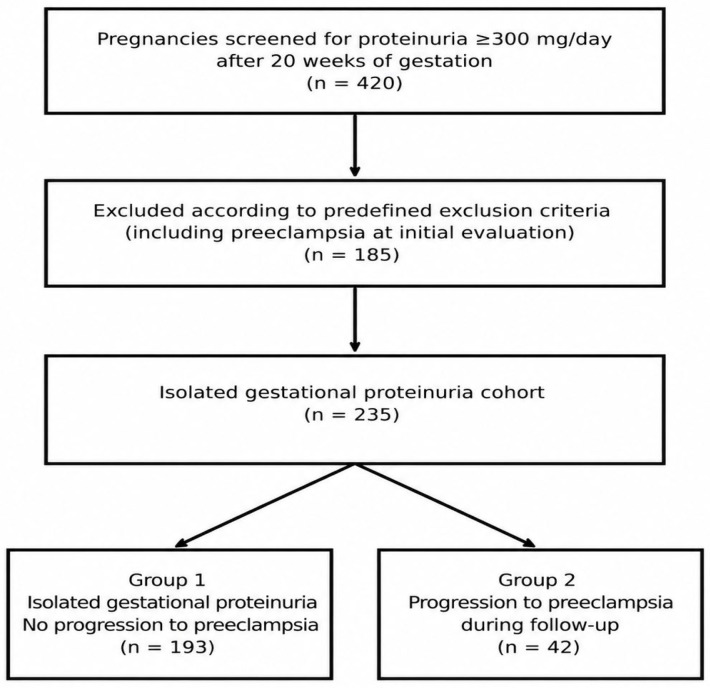
Flowchart of the study.

A total of 420 pregnancies with proteinuria ≥300 mg/day, confirmed by 24 h urine collection after 20 weeks of gestation, were initially screened. Patients who met predefined exclusion criteria, including women who already met the diagnostic criteria for preeclampsia at the initial evaluation due to concomitant hypertension (*n* = 84), were excluded. The remaining isolated gestational proteinuria cohort included 235 pregnancies. The final cohort was then divided into two groups based on progression to preeclampsia during follow-up. Group 1 included women with isolated gestational proteinuria who did not develop hypertension during pregnancy (*n* = 193), while Group 2 consisted of patients whose condition progressed to preeclampsia during follow-up (*n* = 42), representing 17.8% of the final isolated gestational proteinuria cohort. The flowchart of the study population is shown in Figure 1.

### 2.3. Definition of Terms

Isolated gestational proteinuria is defined as significant proteinuria detected after 20 weeks of gestation without hypertension or other diagnostic criteria for preeclampsia at diagnosis and throughout pregnancy follow-up [3]. Gestational hypertension (GH) is defined as a systolic blood pressure of 140 mm Hg or higher, a diastolic blood pressure of 90 mm Hg or higher, or both, after 20 weeks of gestation in a woman with previously normal blood pressure [1,2]. The American College of Obstetricians and Gynecologists (ACOG) defines preeclampsia as new-onset hypertension after 20 weeks of gestation accompanied by either proteinuria or, in the absence of proteinuria, evidence of maternal organ dysfunction such as thrombocytopenia, renal insufficiency, impaired liver function, pulmonary edema, or cerebral or visual symptoms [1].

Ultrasound measurements were performed by staff at the perinatology clinic. Ultrasound examinations used a General Electric Voluson S10 (GE Healthcare, General Electric Company, Chicago, MA, USA) with a 3.5 MHz abdominal convex transducer. Placental dysfunction–related findings were defined as the presence of fetal growth restriction (FGR), small for gestational age (SGA), oligohydramnios, or abnormal umbilical artery Doppler findings such as absent end-diastolic flow. According to the Delphi consensus criteria, fetuses with birth weight below the 3rd percentile are classified as having fetal growth restriction, while those with birth weight between the 3rd and 10th percentiles are classified as small for gestational age [9]. The maximum vertical pocket method is preferred for amniotic fluid assessment during fetal examination. Oligohydramnios is defined as a maximum vertical pocket of less than 2 cm according to the 2014 Fetal Ultrasound Imaging Workshop [10]. Abnormal umbilical artery Doppler findings were defined as absent end-diastolic flow (AEDF) in the umbilical artery waveform according to ISUOG guidelines [11].

Clinical, obstetric, and neonatal data were obtained from the electronic medical record system. Laboratory parameters were recorded at two time points: at the diagnosis of proteinuria and at the time the decision for delivery was made. Laboratory data were collected to assess hematological and systemic inflammation markers (NLR, SIRI, MLR, PLR, and SII). These parameters were calculated using the following formulas: NLR, neutrophil count divided by lymphocyte count; MLR, monocyte count divided by lymphocyte count; SIRI, neutrophil count multiplied by monocyte count divided by lymphocyte count; SII, platelet count multiplied by neutrophil count divided by lymphocyte count; and PLR, platelet count divided by lymphocyte count [12].

### 2.4. Statistical Analysis

Statistical analyses were performed using the Statistical Package for the Social Sciences Version 22.0 (IBM Corporation, Armonk, New York, NY, USA). The distribution of variables was assessed with the Kolmogorov–Smirnov test. As the variables did not follow a normal distribution, continuous variables were reported as medians (interquartile ranges, IQRs) and compared between groups using the Mann–Whitney U test. Categorical variables were presented as numbers and percentages (n, %) and analyzed using the chi-square test or Fisher’s exact test.

Clinically relevant variables and variables demonstrating statistical significance in univariate analyses (*p* < 0.10) were entered into a multivariable logistic regression model to identify independent predictors of progression from isolated gestational proteinuria to preeclampsia. Variables were selected based on baseline statistical significance, together with clinical plausibility and relevance to preeclampsia pathophysiology, rather than automated variable selection procedures. Inflammatory indices included in the analyses were selected based on their previously reported associations with hypertensive disorders of pregnancy in the existing literature and their routine availability in clinical practice. Multicollinearity among independent variables was assessed using the variance inflation factor (VIF) and tolerance values.

Receiver operating characteristic (ROC) curve analysis was used to evaluate the discriminative ability of both individual predictors and the combined multivariable prediction model. Calibration of the multivariable model was assessed using the Hosmer–Lemeshow goodness-of-fit test. A *p*-value < 0.05 was considered statistically significant.

## 3. Results

Maternal demographic characteristics of the study population are shown in Table 1. Maternal age was significantly higher in patients who progressed to preeclampsia compared with those who had isolated proteinuria (median 34 vs. 32 years, *p* = 0.028). Similarly, body mass index was significantly higher in the progression group (34.5 vs. 32.0 kg/m^2^, *p* = 0.010). Gravidity and parity were similar between the two groups.

Baseline maternal clinical and laboratory characteristics at the time of proteinuria diagnosis are shown in Table 2. Gestational age at diagnosis was significantly lower in patients who later developed preeclampsia compared to those with isolated proteinuria (31 vs. 33 weeks, *p* = 0.003). Systolic blood pressure at diagnosis was also higher in the progression group (120 vs. 115 mmHg, *p* = 0.009). Among laboratory parameters, neutrophil count was significantly higher in patients who progressed to preeclampsia (*p* = 0.004). Inflammatory indices, including the systemic immune-inflammation index (SII) and neutrophil-to-lymphocyte ratio (NLR), were significantly elevated in the progression group (*p* = 0.015 and *p* = 0.011, respectively). Additionally, glomerular filtration rate was significantly lower in patients who developed preeclampsia (*p* = 0.037). Other laboratory parameters, including platelet count, lymphocyte count, SIRI, PLR, uric acid, and liver enzymes, were similar between the groups.

Maternal clinical and laboratory findings at delivery are summarized in Table 3. Patients who developed preeclampsia delivered at an earlier gestational age than those with isolated proteinuria (35 vs. 37 weeks, *p* = 0.001). Hemoglobin and hematocrit levels at delivery were significantly higher in the progression group (*p* = 0.003 and *p* = 0.011, respectively). Serum albumin levels were significantly lower in patients with preeclampsia (*p* = 0.021), while alanine aminotransferase (ALT) levels were significantly higher (*p* = 0.006). Systolic and diastolic blood pressures at delivery were significantly higher in the progression group than in the isolated proteinuria group (*p* < 0.001 for both).

Obstetric outcomes were also compared between the groups. Absent end-diastolic flow in the umbilical artery was observed in 24 (12.4%) women in the isolated proteinuria group and 9 (21.4%) women in the group that progressed to preeclampsia, with no statistically significant difference between the groups. The frequencies of oligohydramnios, small for gestational age, and fetal growth restriction were also similar between the groups. Oligohydramnios was observed in 8 (4.1%) patients in the isolated proteinuria group and 3 (7.1%) patients in the progression group. Small for gestational age was detected in 2 (1.0%) cases in the isolated proteinuria group and in none of the patients in the progression group. Fetal growth restriction occurred in 43 (22.3%) pregnancies in the isolated proteinuria group and 11 (26.2%) pregnancies in the progression group (*p* > 0.05 for both) (Table 4).

Neonatal outcomes are shown in Table 5. Birth weight was significantly lower in pregnancies that progressed to preeclampsia compared with the isolated proteinuria group (2220 g vs. 2600 g, *p* = 0.037). Cesarean delivery was significantly more frequent in the progression group (92.9% vs. 68.9%, *p* = 0.001). However, Apgar scores at 1 and 5 min and neonatal intensive care unit (NICU) admission rates were similar between the groups.

Univariate logistic regression analysis identified maternal age, body mass index, gestational age at diagnosis, systolic blood pressure, and neutrophil count at diagnosis as potential predictors of progression from isolated proteinuria to preeclampsia (Table 6). In multivariate logistic regression analysis, higher body mass index (aOR 1.085, 95% CI 1.008–1.167, *p* = 0.028) and earlier gestational age at diagnosis (aOR 0.883, 95% CI 0.802–0.972, *p* = 0.011) remained independent predictors of progression to preeclampsia. Systolic blood pressure and neutrophil count were not statistically significant after adjustment for potential confounders. No evidence of multicollinearity was observed, with all VIF values close to 1.

**Figure 2:**
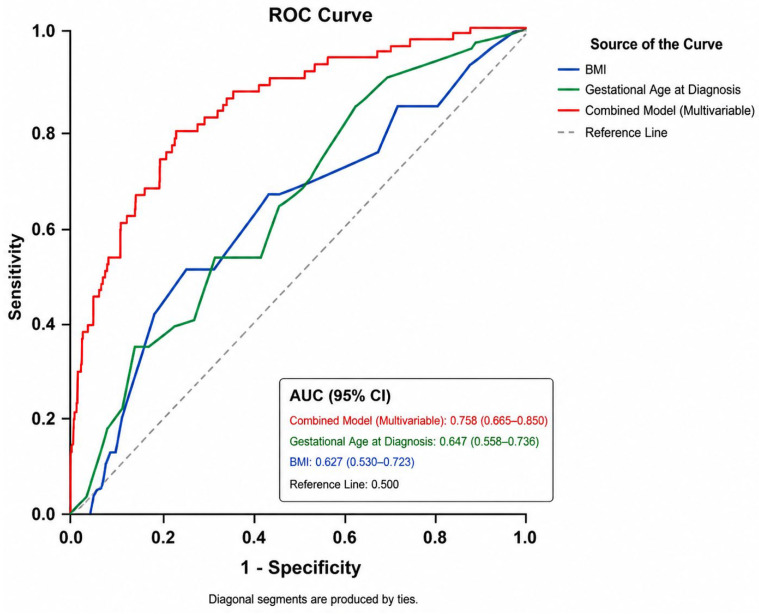
Receiver operating characteristic (ROC) curves of body mass index (BMI) and gestational age at diagnosis for predicting progression to preeclampsia.

Receiver operating characteristic (ROC) curve analysis demonstrated modest but statistically significant discriminative performance for both gestational age at diagnosis and maternal BMI in predicting progression to preeclampsia. Gestational age at diagnosis yielded an AUC of 0.647 (95% CI: 0.558–0.736, *p* = 0.003), and a cutoff value of ≤32 weeks predicted progression with a sensitivity of 63% and specificity of 54%. Similarly, BMI showed an AUC of 0.627 (95% CI: 0.530–0.723, *p* = 0.010), with a cutoff value of 32.5 kg/m^2^ predicting progression with a sensitivity of 64% and specificity of 57%. The ROC curves for gestational age at diagnosis and BMI are shown in Figure 2.

ROC analysis of individual renal parameters showed poor discriminative performance. The AUC values were 0.531 for 24 h proteinuria (*p* = 0.535), 0.477 for BUN (*p* = 0.646), and 0.404 for GFR (*p* = 0.055), indicating no significant predictive value. Combined analysis of these renal parameters did not improve predictive performance (AUC: 0.592, *p* = 0.065).

To further assess the predictive performance of the multivariable model, ROC curve analysis was performed using predicted probabilities from the combined logistic regression model. The combined model showed improved overall discriminative performance compared with individual predictors, with an AUC of 0.758 (95% CI: 0.665–0.850, *p* < 0.001) (Figure 2).

Calibration analysis using the Hosmer–Lemeshow goodness-of-fit test showed acceptable agreement between predicted and observed outcomes (χ^2^ = 7.873, df = 8, *p* = 0.446). The overall multivariable model was statistically significant according to the omnibus test of model coefficients (χ^2^ = 22.694, df = 5, *p* < 0.001). The model explained approximately 20.2% of the variance in progression risk according to the Nagelkerke R^2^ value.

## 4. Discussion

In this study, we evaluated clinical, laboratory, and inflammatory parameters in pregnancies complicated by isolated gestational proteinuria (IGP) and investigated factors associated with progression to preeclampsia. Approximately 17.8% of women with IGP progressed to preeclampsia during follow-up. The key finding of our study is that traditional clinical parameters—particularly maternal body mass index (BMI) and gestational age at diagnosis—were the only independent predictors of progression, while systemic inflammatory indices and renal function parameters did not show independent predictive value after multivariable adjustment.

Our findings indicate that IGP should not be considered a benign condition. The observed progression rate aligns with previous studies reporting rates between 19.6% and 25%, supporting the concept that isolated proteinuria may be an early clinical manifestation of placental dysfunction rather than a transient or incidental finding. In this context, our results underscore the importance of careful clinical surveillance in pregnancies complicated by IGP [4,5,13].

However, despite statistical significance, the discriminative performance of individual predictors remained modest, indicating limited clinical utility for individualized prediction. Although the combined multivariable model showed improved predictive performance compared with individual variables, the overall predictive ability remained moderate. These findings suggest that currently available clinical and laboratory parameters are insufficient for robust prediction of progression from IGP to preeclampsia.

A key finding of this study is the independent association between higher maternal BMI and progression to preeclampsia. Although some previous studies did not find a significant relationship between BMI and progression risk, our results align with the broader literature linking maternal obesity to hypertensive disorders of pregnancy [4,5]. The underlying pathophysiological mechanisms may include endothelial dysfunction, chronic low-grade systemic inflammation, oxidative stress, and impaired placentation [14,15]. These biological processes may increase susceptibility to disease progression among patients who initially present with isolated gestational proteinuria.

However, despite its independent statistical association, the predictive performance of BMI was modest, with limited discriminative ability on its own. Therefore, maternal BMI should not be considered a clinically actionable predictor in isolation, but rather as one component of the overall risk profile for progression to preeclampsia.

Another important finding of our study was that an earlier gestational age at the time of proteinuria diagnosis was independently associated with progression to preeclampsia. Earlier onset of proteinuria may indicate more pronounced placental dysfunction and earlier activation of the pathological processes underlying preeclampsia. This observation supports the hypothesis that isolated gestational proteinuria may represent an early stage within the clinical spectrum of preeclampsia rather than a distinct benign condition.

However, despite statistical significance, the discriminative performance of gestational age at diagnosis remained modest and insufficient for reliable individualized clinical prediction when used alone. These findings further emphasize the limited predictive value of currently available clinical parameters when used alone in pregnancies complicated by isolated gestational proteinuria.

In contrast to clinical parameters, inflammatory indices did not show independent predictive value in our study. This finding is particularly relevant given the growing interest in systemic inflammatory markers as potential predictors of preeclampsia [6,7,8,16]. Our results suggest that, in the specific context of isolated gestational proteinuria, these indices may primarily reflect nonspecific maternal systemic inflammatory activation rather than serve as reliable tools for risk stratification. Accordingly, inflammatory markers did not provide meaningful incremental predictive value beyond conventional clinical parameters in this specific population. Previous studies have similarly reported inconsistent and often limited discriminatory performance of laboratory-derived inflammatory markers in preeclampsia, supporting the concept that systemic inflammatory indices may insufficiently reflect the complex placental and endothelial mechanisms underlying disease progression [17]. The discrepancy between our findings and previous reports may be partly explained by differences in study populations. Most prior studies evaluated inflammatory markers by comparing patients with established preeclampsia to healthy control groups, whereas our study specifically focused on progression to preeclampsia within an already clinically enriched high-risk cohort of women with isolated gestational proteinuria. In this setting, baseline inflammatory activation may already be elevated across the study population, reducing the discriminatory performance of inflammatory indices. Taken together, these findings challenge the growing assumption that laboratory-derived inflammatory biomarkers meaningfully improve the prediction of preeclampsia in all clinical contexts. Our results suggest that inflammatory biomarkers may offer limited additional predictive value beyond conventional clinical risk factors.

Several pathophysiological mechanisms may explain the limited independent predictive value of inflammatory indices observed in our study. First, laboratory-derived inflammatory markers such as NLR, SII, and SIRI may primarily reflect nonspecific maternal systemic inflammatory activity rather than placental dysfunction, which is considered central to the pathogenesis of preeclampsia. Second, pregnancies complicated by isolated gestational proteinuria may already represent a clinically enriched or relatively advanced stage within the spectrum of placental disease. In this context, inflammatory activation may already be present across the cohort, reducing the discriminatory capacity of these markers between patients who subsequently progress to preeclampsia and those who do not. Third, inflammatory indices were assessed at a single time point, whereas the biological processes underlying preeclampsia are dynamic and evolve throughout pregnancy. Therefore, single-measurement inflammatory markers may inadequately capture temporal changes in endothelial dysfunction, placental ischemia, and maternal immune adaptation. Collectively, these mechanisms may partly explain why inflammatory indices failed to demonstrate meaningful independent predictive value after multivariable adjustment in our cohort.

Similarly, renal function parameters, including glomerular filtration rate (GFR), did not show independent predictive value for progression to preeclampsia after multivariable adjustment. Overall, these findings suggest that conventional clinical characteristics may be more informative than laboratory-derived inflammatory or renal indices for predicting progression from isolated gestational proteinuria to preeclampsia. Nevertheless, even the predictive performance of clinical parameters remained modest, highlighting the ongoing limitations of currently available tools for individualized risk prediction in this population.

The clinical implications of these findings indicate that risk assessment in pregnancies complicated by isolated gestational proteinuria should primarily rely on simple, readily available clinical parameters rather than complex inflammatory or biochemical indices. Patients with higher maternal BMI and earlier gestational age at diagnosis may require closer maternal and fetal surveillance, while routine use of inflammatory indices for prediction appears to offer limited additional clinical value.

However, the predictive performance of both clinical and laboratory parameters remained moderate overall, suggesting that currently available tools are insufficient for robust individualized prediction of progression from isolated gestational proteinuria to preeclampsia.

These findings should be interpreted within the context of a clinically selected high-risk population, rather than as reflecting prediction in an unselected obstetric cohort. The nature of the study population may have reduced biological and clinical variability between groups, thereby diminishing the apparent discriminatory performance of inflammatory biomarkers. Therefore, the present findings should be viewed as evaluating prediction within an already high-risk population, rather than assessing screening utility in the general obstetric population.

This study has several strengths. First, it evaluated a well-defined cohort of pregnancies complicated by isolated gestational proteinuria and systematically assessed clinical, laboratory, inflammatory, and renal parameters associated with progression to preeclampsia. Second, the inclusion of both maternal and neonatal outcomes provided a more comprehensive evaluation of the clinical course and prognostic implications of isolated gestational proteinuria.

Additionally, inflammatory indices derived from routinely available laboratory parameters were analyzed, increasing the potential real-world clinical applicability of the findings. Another important strength is that the study specifically evaluated the prediction of progression to preeclampsia within an already clinically enriched high-risk population, rather than simply comparing patients with established preeclampsia to healthy controls. This design allowed a more clinically relevant assessment of whether inflammatory biomarkers provide incremental predictive value beyond conventional clinical parameters in real-world obstetric practice.

This study has several limitations. First, there is potential for selection bias. The study cohort was drawn from a tertiary referral center, and 24 h urine protein assessment was performed based on clinical indications—such as suspected hypertensive disorders or abnormal screening findings—rather than universal screening. Therefore, the analyzed population likely represents a clinically selected higher-risk subgroup rather than the full spectrum of isolated gestational proteinuria seen in general obstetric practice. This introduces the possibility of indication and referral bias, which may have influenced both the observed progression rate and the strength of the identified associations. Consequently, the generalizability of the findings to low-risk or unselected obstetric populations may be limited.

The substantial exclusion process and clinically indicated patient selection strategy resulted in a highly enriched high-risk study population. This composition may have reduced biological and clinical variability between groups and potentially attenuated the apparent discriminatory performance of inflammatory biomarkers. Accordingly, these findings should be interpreted as reflecting prediction within an already clinically high-risk population rather than evaluating screening performance in an unselected obstetric cohort.

Second, the retrospective design inherently carries risks of residual confounding and incomplete control of potential covariates. In addition, the single-center nature of the study may further limit external validity.

Third, systemic inflammatory markers were derived from routinely available blood parameters and assessed at a single time point, which may not adequately capture dynamic inflammatory changes throughout pregnancy. Although several inflammatory indices were evaluated, their predictive value did not persist after multivariable adjustment. This may indicate that these markers primarily reflect nonspecific maternal systemic inflammatory responses rather than robust predictors of disease progression.

Finally, although the number of progression events allowed multivariable modeling, the relatively limited number of events may have affected model stability, increased the risk of overfitting, limited the precision of the estimated associations, and reduced the feasibility of more advanced internal validation techniques. Therefore, extrapolation of these findings to universally screened or lower-risk obstetric populations should be performed cautiously. The predictive performance observed in this tertiary referral cohort may differ from that seen in general obstetric practice.

## 5. Conclusions

In conclusion, isolated gestational proteinuria should not be considered a benign condition and requires careful clinical surveillance, as a substantial proportion of affected pregnancies may progress to preeclampsia. In this cohort, approximately one in six women developed preeclampsia during follow-up. Higher maternal body mass index and earlier gestational age at diagnosis were identified as independent predictors of progression. Progression to preeclampsia was also associated with lower birth weight, earlier delivery, and a higher rate of cesarean delivery.

Our findings suggest that simple and readily available clinical parameters—particularly maternal BMI and gestational age at diagnosis—may be more informative than laboratory-derived inflammatory or renal indices for risk assessment in pregnancies complicated by isolated gestational proteinuria. Women with these clinical risk features may benefit from closer maternal and fetal monitoring.

However, the predictive performance of both clinical and laboratory parameters remained moderate overall, indicating that currently available tools are insufficient for robust individualized prediction of progression from isolated gestational proteinuria to preeclampsia. Importantly, these findings question the routine clinical utility of inflammatory indices as standalone predictive tools in this high-risk population. Therefore, these markers should not be interpreted as clinically actionable discriminators when used alone.

Further large-scale prospective studies with external validation are needed to confirm these findings and to develop more reliable prediction models for progression from isolated gestational proteinuria to preeclampsia.

## Figures and Tables

**Table 1:** Maternal demographic and clinical characteristics of the groups.

Parameter	Isolated Proteinuria (*n* = 193)	Progression to Preeclampsia (*n* = 42)	*p* Value
Age (years)	32.0 (9)	34.0 (10)	*0.028*
Gravidity (n)	2.0 (2.0)	2.0 (2.0)	0.670
Parity (n)	2.0 (2.0)	2.0 (2.0)	0.659
BMI at diagnosis (kg/m^2^)	32.0 (8.0)	34.5 (10.0)	*0.010*

BMI: body mass index. Data are presented as median [IQR] unless otherwise specified. Statistically significant values are presented in italics.

**Table 2:** Baseline maternal clinical and laboratory characteristics at diagnosis of the groups.

Parameter	Isolated Proteinuria (*n* = 193)	Progression to Preeclampsia (*n* = 42)	*p* Value
**Clinical parameters**			
Gestational age (weeks)	33.0 (6.0)	31.0 (8.0)	*0.003*
24 h proteinuria (mg)	461 (380)	531 (1098)	0.501
24 h urine volume (cc)	2515 (1445)	2400 (1400)	0.364
Systolic blood pressure (mmHg)	115.0 (17.0)	120.0 (15.0)	*0.009*
Diastolic blood pressure (mmHg)	70.0 (11.0)	72.0 (11.0)	0.392
**Hematologic parameters**			
Hemoglobin (g/dL)	11.5 (1.8)	12.4 (1.8)	0.059
Hematocrit (%)	35.6 (4.9)	36.2 (4.4)	0.647
White blood cell count (×10^3^/µL)	7.1 (7.8)	5.5 (8.5)	0.919
Platelet count (×10^3^/µL)	244.0 (94.5)	234.0 (76.0)	0.722
Neutrophil count (×10^3^/µL)	6.1 (3.2)	7.3 (2.9)	*0.004*
Lymphocyte count (×10^3^/µL)	1.9 (0.9)	1.7 (0.9)	0.155
Monocyte count (×10^3^/µL)	0.6 (0.3)	0.6 (0.4)	0.110
SII	679.6 (686.1)	842.2 (786.6)	*0.015*
SIRI	1.86 (1.7)	2.03 (1.9)	0.331
NLR	2.98 (2.2)	3.86 (3.43)	*0.011*
MLR	0.33 (0.17)	0.30 (0.18)	0.219
PLR	124.6 (71.7)	128.4 (70.5)	0.274
**Renal/hepatic parameters**			
BUN (mg/dL)	16.7 (8.0)	17.7 (7.0)	0.586
Creatinine (mg/dL)	0.54 (0.19)	0.55 (0.15)	0.377
GFR	127.0 (15.0)	121.5 (17.0)	*0.037*
Uric acid (mg/dL)	4.6 (1.8)	4.9 (2.7)	0.489
Albumin (g/L)	35.0 (4.8)	34.5 (4.3)	0.997
Total protein (g/L)	61.4 (6.9)	59.9 (8.8)	0.607
ALT (U/L)	11.0 (7.0)	11.0 (9.0)	0.859
AST (U/L)	16.0 (10.0)	16.0 (11.0)	0.784

NLR: neutrophil-to-lymphocyte ratio; MLR: monocyte-to-lymphocyte ratio; PLR: platelet-to-lymphocyte ratio; SII: systemic immune-inflammation index; and SIRI: systemic inflammation response index, GFR: glomerular filtration rate. Data are presented as median [IQR] unless otherwise specified. Statistically significant values are presented in italics.

**Table 3:** Maternal clinical and laboratory characteristics at delivery of the groups.

Parameter	Isolated Proteinuria (*n* = 193)	Progression to Preeclampsia (*n* = 42)	*p* Value
**Clinical parameters**			
Gestational age (weeks)	37.0 (4.0)	35.0 (5.0)	*0.001*
Systolic blood pressure (mmHg)	124.0 (16.0)	147.5 (15.0)	<*0.001*
Diastolic blood pressure (mmHg)	76.0 (10.0)	90.0 (19.0)	<*0.001*
**Hematologic parameters**			
Hemoglobin (g/dL)	11.9 (1.6)	12.7 (1.4)	*0.003*
Hematocrit (%)	36.5 (4.7)	38.3 (4.5)	*0.011*
Platelet count (×10^3^/µL)	236.0 (94)	222.0 (92)	0.341
Neutrophil count (×10^3^/µL)	6.0 (6.0)	6.5 (4.3)	0.233
Lymphocyte count (×10^3^/µL)	1.8 (0.8)	2.0 (0.8)	0.392
Monocyte count (×10^3^/µL)	0.6 (0.2)	0.6 (0.2)	0.894
SII	620.4 (702.3)	548.8 (748.5)	0.923
SIRI	1.71 (2.0)	2.04 (2.3)	0.401
NLR	2.76 (3.13)	3.00 (3.22)	0.377
MLR	0.35 (0.18)	0.33 (0.18)	0.331
PLR	124.0 (66.8)	112.7 (73.8)	0.079
**Renal/hepatic parameters**			
BUN (mg/dL)	19.0 (10.8)	20.1 (17.8)	0.263
Creatinine (mg/dL)	0.57 (0.27)	0.63 (0.27)	0.061
Uric acid (mg/dL)	5.3 (2.2)	5.7 (2.8)	0.136
Albumin (g/L)	34.3 (5.6)	32.3 (6.6)	*0.021*
Total protein (g/L)	60.0 (7.2)	59.0 (11.8)	0.066
ALT (U/L)	11.0 (7.0)	14.0 (11.0)	*0.006*
AST (U/L)	17.0 (8.0)	18.0 (17.0)	0.106

NLR: neutrophil-to-lymphocyte ratio; MLR: monocyte-to-lymphocyte ratio; PLR: platelet-to-lymphocyte ratio; SII: systemic immune-inflammation index; and SIRI: systemic inflammation response index. Data are presented as median [IQR] unless otherwise specified. Statistically significant values are presented in italics.

**Table 4:** Comparison of placental dysfunction–related obstetric outcomes between pregnancies with isolated proteinuria and those progressing to preeclampsia.

Parameter	Isolated Proteinuria (*n* = 193)	Progression to Preeclampsia (*n* = 42)	*p* Value
Absent end-diastolic flow in the umbilical artery Doppler (n, %)	24 (12.4%)	9 (21.4%)	0.128
Oligohydramnios (n, %)	8 (4.1%)	3 (7.1%)	0.419
Small for gestational age (n, %)	2 (1.0%)	0 (0.0%)	1.000
Fetal growth restriction (n, %)	43 (22.3%)	11 (26.2%)	0.585

**Table 5:** Delivery and neonatal outcomes in women with isolated proteinuria and those who subsequently developed preeclampsia.

Parameter	Isolated Proteinuria (*n* = 193)	Progression to Preeclampsia (*n* = 42)	*p* Value
Birth weight (g) (median, IQR)	2600 (1200)	2220 (1735)	*0.037*
Mode of delivery Cesarean section (n, %) Vaginal delivery (n, %)	133 (68.9%) 60 (31.1%)	39 (92.9%) 3 (7.1%)	*0.001*
1 min Apgar score (median, IQR)	8.0 (2.0)	9.0 (2.0)	0.876
5 min Apgar score (median, IQR)	9.0 (2.0)	10.0 (2.0)	0.805
NICU admission (n, %)	57 (29.7%)	16 (38.1%)	0.287

IQR: interquartile range; NICU: neonatal intensive care unit. Statistically significant values are presented in italics.

**Table 6:** Univariate and multivariable logistic regression analysis of clinically relevant baseline variables.

		Univariate			Multivariate	
Parameter	OR	95% CI	*p* Value	aOR	95% CI	*p* Value
Age	1.066	1.007–1.129	*0.028*	1.020	0.943–1.102	0.622
BMI	1.070	1.013–1.130	*0.015*	1.085	1.009–1.167	*0.028*
Gestational age	0.886	0.819–0.959	*0.003*	0.883	0.802–0.972	*0.011*
Systolic blood pressure	1.048	1.009–1.088	*0.015*	1.036	0.996–1.078	0.076
GFR	0.983	0.965–1.002	0.072	0.995	0.960–1.030	0.762
SII	1.013	0.984–1.043	0.383	-	-	-
NLR	1.099	0.982–1.230	0.102	-	-	-
Neutrophil count (per 1000 cells)	1.171	1.029–1.333	*0.017*	1.156	0.991–1.348	0.064

NLR: neutrophil-to-lymphocyte ratio; BMI: body mass index SII: systemic immune-inflammation index; OR: odds ratio; aOR: adjusted odds ratio; CI: confidence interval. Statistically significant values are presented in italics.

## Data Availability

The datasets used and/or analyzed during the current study are available from the corresponding author on reasonable request.
